# Molecular polymorphisms of the nuclear and chloroplast genomes among African melon germplasms reveal abundant and unique genetic diversity, especially in Sudan

**DOI:** 10.1093/aob/mcaf028

**Published:** 2025-04-17

**Authors:** Odirichi Nnennaya Imoh, Gentaro Shigita, Mitsuhiro Sugiyama, Tran Phuong Dung, Katsunori Tanaka, Mami Takahashi, Kazusa Nishimura, Yuki Monden, Hidetaka Nishida, Mashaer Goda, Michel Pitrat, Kenji Kato

**Affiliations:** Graduate School of Environmental and Life Science, Okayama University, Okayama, 700-8530, Japan; Graduate School of Environmental and Life Science, Okayama University, Okayama, 700-8530, Japan; Department of Life Science Systems, Technical University of Munich, Freising 85354, Germany; Institute of Vegetable and Floriculture Science, National Agriculture and Food Research Organization (NARO), Mie 514-2392, Japan; Graduate School of Environmental and Life Science, Okayama University, Okayama, 700-8530, Japan; Faculty of Agriculture and Life Science, Hirosaki University, Aomori, 036-8561, Japan; Graduate School of Environmental and Life Science, Okayama University, Okayama, 700-8530, Japan; Graduate School of Environmental, Life, Natural Science and Technology, Okayama University, Okayama, 700-8530, Japan; Graduate School of Environmental, Life, Natural Science and Technology, Okayama University, Okayama, 700-8530, Japan; Graduate School of Environmental, Life, Natural Science and Technology, Okayama University, Okayama, 700-8530, Japan; Plant Genetic Resources Conservation and Research Center, Agricultural Research Corporation, Wadmadani, Sudan; INRAE, UR1052, Génétique et amélioration des fruits et légumes, 84143 Montfavet cedex, France; Graduate School of Environmental, Life, Natural Science and Technology, Okayama University, Okayama, 700-8530, Japan

**Keywords:** *Cucumis melo*, Africa, chloroplast genome, domestication, genetic diversity, genetic resources, maternal lineage, melon, phylogeny, polyphyletic origin, seed size, Tibish

## Abstract

**Background and Aims:**

Africa is rich in wild species of *Cucumis* and is considered one of the places of origin of melon. However, our knowledge of African melon is limited, and genetic studies using melon germplasms with wide geographical coverage are required. Here, we analysed the genetic structure of African melons, with emphasis on Sudan.

**Methods:**

Ninety-seven accessions of African melon were examined along with 77 reference accessions representing Asian melon and major horticultural groups. Molecular polymorphisms in the nuclear and chloroplast genomes were investigated using 12 RAPD, 7 SSR and 3 SNP markers. Horticultural traits, including seed size, were measured for 46 accessions, mainly from Sudan.

**Key Results:**

African melons were divided into large and small seed-types based on seed length: large seed-type from Northern Africa and small seed-type from Western and Southern Africa. Both seed types are common in Sudan. Molecular genetic diversity in these geographical populations was as high as in India, the Asian centre of melon domestication. Large seed-types from Northern Africa were assigned to Pop4 by structure analysis and had Ib cytoplasm in common with Cantalupensis, Inodorus and Flexuosus. Small seed-types were highly diversified and geographically differentiated; specifically, Pop1 with Ia cytoplasm in Southern Africa and South Asia, Pop2 with Ia in East Asia, including Conomon and Makuwa, and Pop3 with Ia or Ic in Africa. Sudanese small seed-types were grouped in Pop3, while their cytoplasm type was a mixture of Ia and Ic. Sudanese Tibish had Ic cytoplasm, which was unique in Africa, common in Western Africa and Sudan, and also found in wild or feral types.

**Conclusions:**

Melon of Ic lineage, including Tibish, originated from wild melon in the ‘western Sudan region’, and independently of melon with Ia or Ib cytoplasm, which originated in Asia. This clearly indicates the polyphyletic origin of melon.

## INTRODUCTION

The genus *Cucumis* is one of the most diversified genera in the Cucurbitaceae family (Schaefer and Renner, 2011). Melon (*Cucumis melo*) is a horticulturally important vegetable cultivated in most tropical to temperate regions (Eduardo *et al.*, 2007; Szamosi *et al.*, 2010). Melon has great morphological and physiological variations in size, taste, aroma and colour of fruit, and flower type, which has made its classification controversial (Laghetti *et al.*, 2008; Escribano and Lázaro, 2009; Nasrabadi *et al.*, 2012).

Melon is largely categorized into two subspecies, *melo* and *agrestis*, and 19 horticultural groups (Jeffrey, 1990; Kirkbride, 1993; Pitrat, 2016). Horticultural groups defined by Pitrat (2016) are used hereafter. Earlier studies reported Africa and Asia as the likeliest centres of origin for melon, with a secondary centre of diversity in the Middle East (Robinson and Decker-Walters, 1997; Díaz *et al.*, 2017). Based on Sanger sequencing of seven regions of the nuclear and chloroplast genomes, Endl *et al.* (2018) reported that melon was domesticated at least twice in Africa and Asia and found a close wild relative of melon in India (*C. trigonus*). Several other studies also highlighted the richness of genetic diversity in Indian melon by using high-throughput sequencing technology (Gonzalo *et al.*, 2019; Zhao *et al.*, 2019; Liu *et al.*, 2020; Wang *et al.*, 2021; Shigita *et al.*, 2023).

Melon seed size, a quantitative trait, shows continuous variation in a segregating population (Zhang *et al.*, 2022) and among cultivars (Tanaka *et al.*, 2025). This trait is an important criterion reflecting geographical and genetic differentiation among a wide spectrum of melon groups. Based on seed length, melon can be categorized into large seed-type (≥9.0 mm) and small seed-type (<9.0 mm) (Fujishita, 1983; Akashi *et al.*, 2002). Melon groups Cantalupensis and Inodorus are generally classified as large seed-types, and Agrestis, Conomon and Makuwa as small seed-types. Flexuosus, Dudaim and Momordica of South and East Asia have both seed-types. Both seed-types are also distributed in Northern, Western, and Southern Africa (Tanaka *et al.*, 2013, 2024).

Molecular and morphological studies conducted on melon accessions from different parts of Africa remain underexplored. Genetic diversity among African melon germplasms was analysed using random amplified polymorphic DNA (RAPD) markers (Mliki *et al.*, 2001) and simple sequence repeat (SSR) markers (Serres-Giardi and Dogimont, 2012). Trimech *et al.* (2013) and Koubala *et al.* (2016) assessed the morphology and physiochemical properties of the Tunisian and Cameroonian melon of Flexuosus and Tibish, respectively. Imen *et al.* (2015) showed genetic diversity among Tunisian melon landraces of ssp. *agrestis* and Flexuosus using SSR markers, whereas Koffi *et al.* (2014) used amplified fragment length polymorphism (AFLP) markers to determine polymorphisms in melon landraces from Côte d’Ivoire.

Sudan is known to possess highly diverse genetic resources of melon, including both cultivated and wild melon (El-Tahir and Yousif, 2004). Wild melon of ssp. *agrestis*, which is known locally as Humaid, grows naturally in Sudan. For cultivated melon, the four groups in Sudan are Cantalupensis (known locally as Shammam), Flexuosus (Ajjour) and ssp. *agrestis* (Tibish and Seinat). Tibish is eaten as a vegetable in salads or added to cooked meals, while Seinat is eaten for its roasted seeds. Sudanese accessions of Tibish and Agrestis have also been discovered to possess resistance to WCSV (*Watermelon chlorotic stunt virus*), CABYV (*Cucurbit aphid-borne yellow virus*), ZYMV (*Zucchini yellow mosaic virus*), powdery mildew and fusarium wilt (Mohamed *et al.*, 1994; Taha, 2002). Therefore, Sudanese melon genetic resources are indispensable for studying melon genetic diversity and domestication in Africa, as well as for practical melon breeding. However, little is known about genetic diversity in Sudanese melon.

In comparison with the nuclear genome, the chloroplast genome is considered conserved in the gene structure and composition (Asaf *et al.*, 2017), and thus it can provide a better understanding of the relationship between rather distantly related genera or species (Li *et al.*, 2017; Amiryousefi *et al.*, 2018). The polymorphisms embedded in the chloroplast genome can be important in the development of stable markers to study phylogenetics and population genetics (Ahmed *et al.*, 2013; Sheng *et al.*, 2021).

Chung *et al.* (2006), analysing 18 accessions of *Cucumis* species using consensus chloroplast SSR (ccSSR) markers, revealed that cucumber and melon are distinct from African wild species of *Cucumis*. Tanaka *et al.* (2013, 2024) sequenced nine inter- and intragenic regions of the chloroplast genome and revealed the presence of three cytoplasm types (Ia, Ib and Ic) in Africa, thus firmly supporting the African origin of Ic type melon and highlighting the importance and uniqueness of germplasms from Africa.

In this study, we analysed horticultural traits, including seed size, and molecular polymorphisms using 97 African melon germplasms with emphasis on Sudanese melon, and compared them with reference accessions from Europe and Asia. Based on the genetic population structure of the nuclear genome and molecular classification of the chloroplast genome, we aimed to reveal the genetic diversification of African melon and to discuss the African origin of melon.

## MATERIALS AND METHODS

### Plant materials

A total of 174 accessions of melon were examined. Since many of the landraces were not classified into proper horticultural groups, African accessions were divided into four geographical groups to reveal geographical difference in genetic structure, being 38 from Northern Africa, 38 from Sudan, 12 from Western Africa, 9 from Southern Africa, and 97 in total (Table 1, Supplementary Data Table S1). As references we used 77 accessions, which included 31 accessions comprising Flexuosus (2), Cantalupensis (10), Inodorus (5), Conomon (4), Makuwa (5) and Agrestis (5). The remaining 46 reference accessions consisted of three geographical groups of landraces, i.e. India (33), Myanmar (6) and Vietnam (7), and were used for the analysis of genetic relationship among African and Asian melons and for the comparison of genetic diversity. Two accessions of wild cucumber (*Cucumis sativus* var. *hardwickiii*) were used as outgroups. The length and width of seeds were measured for all accessions, and they were classified into small seed-type (<9.0 mm) and large seed-type (≥9.0 mm), according to Akashi *et al*. (2002). These accessions were sourced from Okayama University, Japan; the National Research Institute for Agriculture, Food and Environment (INRAE), France; the National Agriculture and Food Research Organization (NARO) Genebank, Japan; the USDA National Plant Germplasm System, USA; and the Institute of Plant Genetics and Crop Plant Research (IPK), Germany (Supplementary Data Table S1).

**Table 1.** Number of melon accessions studied for each geographical or horticultural group and their classification by seed size.

Region/group	No. of accessions		
	Total	Large seed-type	Small seed-type
African accessions			
Northern Africa	38	37	1
Sudan	38	17	21
Western Africa	12	2	10
Southern Africa	9	1	8
Reference accessions			
Flexuosus	2	2	–
Cantalupensis	10	10	–
Inodorus	5	5	–
India	33	19	14
Myanmar	6	–	6
Vietnam	7	–	7
Conomon	4	–	4
Makuwa	5	–	5
Agrestis	5	–	5
*C. sativus* var. *hardwickii*	2	–	2
Total	176	93	83

### DNA extraction

Cotyledons from a single seedling of each accession were collected 2 weeks after sowing and individually ground in liquid nitrogen. Using the cetyl-trimethyl-ammonium bromide method, total DNA was extracted after Murray and Thompson (1980), with minor modifications. The quality and quantity of each DNA sample were determined using a spectrophotometer.

### Evaluation of horticultural traits

Two plants each of 46 accessions of African melon, including Sudan (36), Northern Africa (7) and Western Africa (3), were grown in the greenhouse of the Institute of Vegetable and Floriculture Science, NARO, Japan, from September 2012 and the following 16 traits were recorded: seed traits (length and width); traits of the 10th leaf (length, width, and petiole length); length of 10th–15th internode; flower type; and fruit traits (weight, length, diameter, flesh thickness, flesh colour, colour and dots of exocarp, fruit groove, and total soluble solids content). Cluster analysis and principal component analysis (PCA) were conducted using these data. Because the variance of fruit weight was much larger than that of the other traits, the data of 16 horticultural traits were standardized to avoid overestimation of the contribution by fruit weight.

### RAPD marker analysis

Ten random primers (12-mer, Bex, Tokyo, Japan; Supplementary Data Table S2) were used for RAPD analysis. Duong *et al.* (2021) selected these primers for their higher reproducibility from 18 primers that had been selected among 176 primers for their ability to detect clear polymorphism among five accessions of ssp. *melo* and ssp. *agrestis* and for reproducibility by Tanaka *et al.* (2007). Twelve marker bands were selected among a total of 87 bands amplified by ten primers for distinct polymorphism and reproducibility. The chromosomal location of the RAPD markers is summarized in Supplementary Data Table S2.

The RAPD marker bands were amplified by PCR in the following conditions: a total of 10 μL mixture, which contained 1.0 μL PCR buffer (Sigma, St Louis, MO, USA: 10 mm Tris–HCl; pH 8.3, 50 mm KCl), 2.5 mm MgCl_2,_ 0.1 mm dNTP, 0.5 μm for each primer, 50 ng genomic DNA, and 0.25 U *Taq* polymerase (Sigma, St Louis, MO, USA). An i-Cycler (Bio-Rad, Hercules, CA, USA) was used in the amplification of the reaction. The PCR cycling profile involved an initial denaturing step at 95 °C for 3 min, 40 cycles at 93 °C for 1 min, 40 °C for 2 min and 72 °C for 2 min. The final extension step was at 72 °C for 5 min. The PCR product was electrophoresed on a 1.5 % agarose gel (GenePure LE, BM Bio, Tokyo, Japan) at a constant voltage of 100 V using a horizontal gel electrophoresis system (Mupid-2, Cosmo Bio, Tokyo, Japan). Ethidium bromide was used for staining the gels, which were then visualized by illumination with UV light.

### SSR marker analysis

Seven SSR markers were used (Supplementary Data Table S2). Duong *et al.* (2021) selected these markers for their higher polymorphism from 16 markers which were selected among 177 markers, developed by Ritschel *et al.* (2004) and Fukino *et al.* (2007), for their ability to detect polymorphism among four accessions of ssp. *melo* and ssp. *agrestis* by Aierkin *et al.* (2011). As summarized in Supplementary Data Table S2, 7 SSR and 12 RAPD markers covered 9 of 12 melon chromosomes. The PCR mixture was the same as for the RAPD analysis described above, but the concentration of the primers was changed to 0.25 μm for each primer. The PCR cycle involved an initial denaturing step at 95 °C for 3 min, 35 cycles at 95 °C for 1 min, 56 °C for 1 min and 72 °C for 2 min. The final extension step was at 72 °C for 5 min. Electrophoresis with 10 % non-denatured polyacrylamide gel at a constant voltage of 260 V was adopted.

### Chloroplast marker analysis

To detect diagnostic SNPs in the chloroplast genome, three markers developed by Tanaka *et al.* (2024) were employed (Supplementary Data Table S3). The chloroplast genome sequence, including SNP, was amplified as follows: a 15 μL mixture containing 50 ng genomic DNA, 1.5 μL PCR buffer (Sigma, St Louis, MO, USA: 10 mm Tris–HCl; pH 8.3, 50 mm KCl), 1.5 mm MgCl_2_, 0.6 mm dNTP, 0.375 μm of each primer and 0.25 U *Taq* polymerase (Sigma, St Louis, MO, USA). Amplification reactions were performed using a T100™ Thermal Cycler (Bio-Rad, Hercules, CA, USA). The PCR cycle involved an initial denaturing step at 95 °C for 3 min, 35 cycles at 95 °C for 1 min, 54 °C for 1.5 min (0.5 min for Cp11-CAPS2), and 72 °C for 2 min. The final extension step was at 72 °C for 5 min. The PCR products of each marker were digested with restriction enzymes, as shown in Supplementary Data Table S3. The process of electrophoresis was the same as for RAPD described above, except for the use of 3 % agarose gel.

### Data analysis

DNA fragments were scored as present (1) and absent (0) for each RAPD marker band (Supplementary Data Fig. S1A), and 1/1 and 0/0 were input as genotype data, respectively. The SSR marker fragments were scored according to their size, from the smallest (1) to the largest (17) as shown in Supplementary Data Fig. S1B, and genotype data were input like 2/2 (homozygote), 3/7 (heterozygote) and so on. SNP markers of the chloroplast genome were genotyped based on the length of the digested DNA fragment, and the cytoplasm type was determined after Tanaka *et al.* (2024) (Supplementary Data Table S4). Genetic similarities (GS) among the accessions were calculated, integrating RAPD and SSR data, by the formula GS = (N11 + N00)/19, where N11 and N00 are the numbers of positive and null bands, respectively, shared between two accessions, and 19 is the total number of marker bands (12 RAPD and 7 SSR), as described by Apostol *et al.* (1993). For SSR markers, the specific products were successfully amplified by all primers in all accessions and thus N00 = 0. In the case of heterozygotes, the number of SSR marker bands shared between two accessions, for example 2/2 and 2/3, will be 0.5. Genetic distance (GD) was calculated using the formula GD = 1 − GS. The polymorphic information content (PIC) of each marker, gene diversity (*D*) within each group, and GD among groups were calculated according to Botstein *et al.* (1980), Weir (1996) and Nei (1972), respectively. A dendrogram was constructed by the PHYLIP program using the unweighted pair-group method with arithmetic average (UPGMA). To show the multiple dimensions of the accessions on a scatter plot, principal coordinate analysis was carried out.

The model-based clustering program STRUCTURE v2.3.4 (Pritchard *et al.*, 2000) was used to infer population structure using a Bayesian approach from the RAPD and SSR marker data sets. The optimal value of *K* (the number of clusters) was deduced by evaluating *K* = 1 − 10 and determined by an admixture model with an allele frequency correlated model. The length of burn-in of the Markov chain Monte Carlo (MCMC) iterations was set to 5000 and data were collected over 5000 MCMC iterations in each run. Twenty iterations per *K* were conducted. The optimal value of *K* was identified using the *ad hoc* procedure introduced by Pritchard *et al.* (2000) and the method developed by Evanno *et al.* (2005), which was carried out in Structure Harvester (Earl and vonHoldt, 2012). Data plotting after the STRUCTURE simulation was conducted with CLUMPP (Jakobsson and Rosenberg, 2007). Accessions with an estimated membership of >0.6 were assigned to the respective populations.

## RESULTS

### Characterization based on horticultural traits

Reference accessions of Cantalupensis, Inodorus and Flexuosus were all large seed-type (≥9.0 mm), while those of Conomon, Makuwa and Agrestis were all small seed-type (<9.0 mm) (Table 1). Of the 97 accessions of African melon, 57 were classified as large seed-type and 40 as small seed-type. The distribution area was different among the two types: large seed-type was common in Northern Africa, while small seed-type was frequent in Western and Southern Africa. In contrast, both types were common in Sudan: 17 and 21 accessions, respectively.

**Fig. 1.**
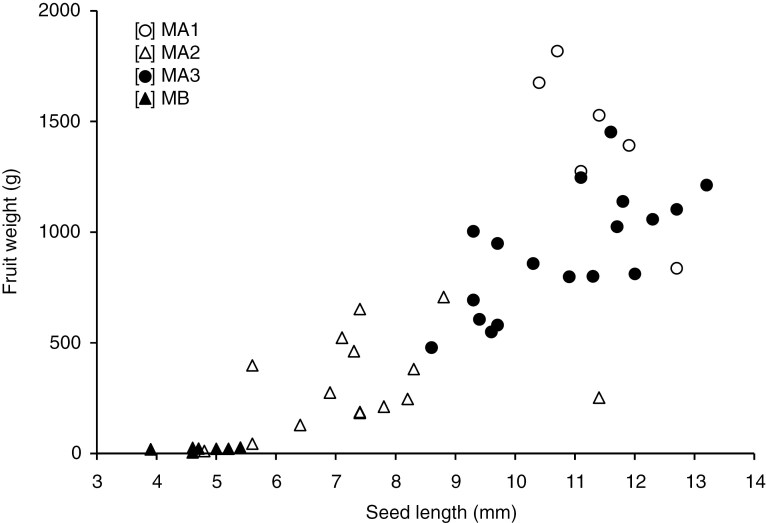
Variation in seed length and fruit weight among 46 accessions of African melon and their difference among four morphological clusters.

**Fig. 2.**
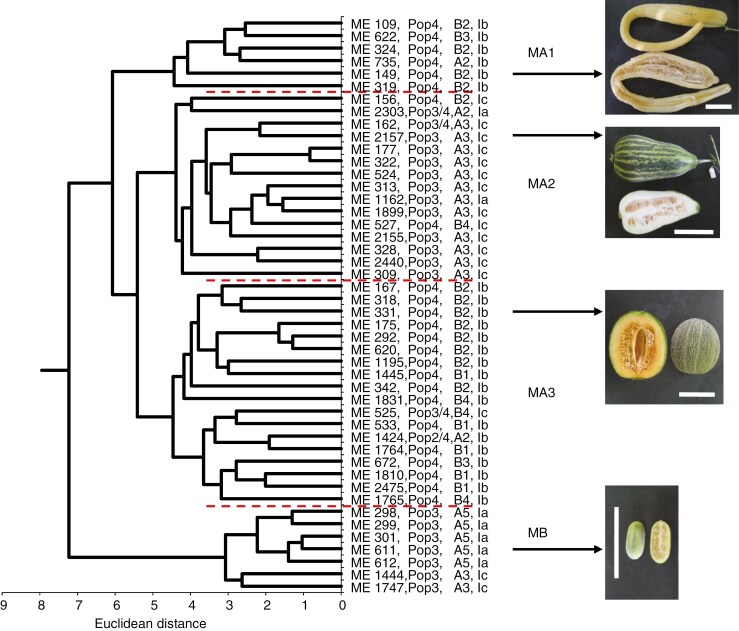
Morphological similarity among 46 accessions of African melon, revealed by cluster analysis. Photographs of mature fruits of representative accessions of four morphological groups are shown on the right. Scale bar is 10 cm. Population (Fig. 3), cluster (Fig. 3) and cytoplasm type (Table 6) of each accession are indicated after the accession number.

Fruit weight varied from 7 g (ME 299) to 1820 g (ME 622) among the 46 accessions examined, and accessions with larger seeds tended to have larger fruits (Fig. 1, *r* = 0.823, *P* < 0.01). Fruit length ranged from 3.4 cm (ME 299) to 81.3 cm (ME 149), and the average of five accessions of Flexuosus was 46.6 cm. African landraces were divided into two major clusters (MA and MB), and cluster MA was further divided into three subclusters (MA1, MA2 and MA3) (Fig. 2). Seven accessions of Agrestis from Sudan (6) and Nigeria (1), classified into cluster MB, commonly had small seeds and small fruits (<30 g) and were also characterized by a monoecious flower type (Table 2).

**Table 2.** Average performance of African melon accessions classified into four groups by cluster analysis based on morphological characters.

Cluster	No. of accessions	Seed length (mm)	Seed width (mm)	Flower type^1^	Fruit weight (g)	Fruit height (cm)	Fruit diameter (cm)
MA1	6	11.4^c^	4.5^bc^	0.5^a^	1422.1^c^	46.5^c^	9.0^bc^
MA2	15	7.4^b^	3.7^b^	1.9^b^	311.8^a^	11.5^ab^	6.7^b^
MA3	18	10.8^c^	4.8^c^	1.9^b^	910.2^b^	14.5^b^	11.3^c^
MB	7	4.8^a^	2.4^a^	0.0^a^	20.9^a^	4.8^a^	2.8^a^

Mean values with different letters indicate significant differences at 0.01 level, by Tukey test.

^1^Scored as 0, monoecious; 2, andromonoecious; and 1, segregating.

**Table 6.** Geographical variation of chloroplast genome type in large and small seed-type melon

	No. of accessions of each chloroplast genome type								
Region/group	Total	Large seed-type				Small seed-type			
		Ia	Ib	Ic	III	Ia	Ib	Ic	III
African accessions									
Northern Africa	38	–	37	–	–	–	–	1	–
Sudan	38	–	15	2	–	7	–	14	–
Western Africa	12	1	1	–	–	–	1	9	–
Southern Africa	9	–	1	–	–	6	–	2	–
Reference accessions									
Flexuosus	2	–	2	–	–	–	–	–	–
Cantalupensis	10	–	10	–	–	–	–	–	–
Inodorus	5	–	5	–	–	–	–	–	–
India	33	9	10	–	–	11	3	–	–
Myanmar	6	–	–	–	–	6	–	–	–
Vietnam	7	–	–	–	–	7	–	–	–
Conomon	4	–	–	–	–	4	–	–	–
Makuwa	5	–	–	–	–	5	–	–	–
Agrestis	5	–	–	–	–	5	–	–	–
*C. sativus* var. *hardwickii*	2	–	–	–	–	–	–	–	2
Total	176	10	81	2	0	51	4	26	2

The remaining 39 accessions were grouped into cluster MA. Subcluster MA1 comprised six accessions of Flexuosus from Sudan (5) and Tunisia (1), which commonly had large seeds and large and long fruits (Table 2). Subcluster MA2 consisted of 15 accessions from Sudan, of which 7 and 5 were classified as Tibish and Agrestis, respectively, and the remaining were unclassified in the passport data. Most of the MA2 accessions had small seeds and rather small fruits, and were also characterized by andromonoecious flower type.

Subcluster MA3 included 18 accessions from Sudan (10), Tunisia (4), Egypt (2), Senegal (1), and Nigeria (1), of which some were classified as Cantalupensis (3), Inodorus (2) and Ameri (2), according to the passport data. These commonly had large seeds, large fruits and a higher Brix value (9.0), and were also characterized by andromonoecious flower type. Of the nine accessions with orange-coloured flesh, eight belonged to subcluster MA3. Photographs of the mature fruits of representative accessions of the four clusters are shown in Fig. 2.

The classification into the above four groups was also reproduced on the PCA plot, in which PC1 and PC2 explained 46.8 % and 15.7 % of the total variance, respectively (Supplementary Data Fig. S2). The MA1 subcluster was differentiated from the other three clusters by the PC1 axis. On the PC2 axis, large seed-type accessions of subclusters MA1 and MA3 were scattered on the right half, while small seed-type accessions of subclusters MA2 and MB were scattered on the left half. This result showed similarity in horticultural traits between Agrestis (MB) and Tibish (MA2) endemic to Sudan, except for flower type, which placed Tibish in cluster MA.

### Analysis of nuclear genome markers

All of the RAPD and SSR markers proved to be polymorphic in 97 accessions of African melon (Supplementary Data Fig. S1), and the PIC of RAPD markers ranged from 0.117 (B32–900 bp) to 0.500 (B71–1220 bp and B99–1400 bp) (Supplementary Data Table S2). The number of alleles detected by SSR markers ranged from 7 (CMBR 120) to 17 (CMN 61–44) and PIC from 0.705 (CMN 04–07) to 0.903 (CMN 61–44).

Gene diversity (*D*) calculated by RAPD and SSR data ranged from 0.413 (Northern Africa) to 0.474 (Sudan) among the four geographical groups of African melon and was equivalent to that of Indian landraces (0.464) (Table 3). Similar results were observed by calculating *D* based solely on the RAPD or SSR data. In contrast, it was ≤0.310 in the four reference groups: Cantalupensis, Inodorus, Conomon and Makuwa. Interestingly, *D* was 0.235 and 0.438 in large seed-type and small seed-type of Sudanese landraces, respectively, being higher in small seed-type. These results indicated that African melon (especially Sudanese melon) was diversified as much as Indian melon analysed in this study.

**Table 3.** Gene diversity calculated based on RAPD and SSR data and number of accessions classified into 12 subclusters shown in Fig. 3.

Region/group	No. of accessions		Gene diversity					No. of accessions of each cluster								
		R + S^1^	RAPD	SSR	A1	A2	A3	A4	A5	B1	B2	B3	B4	B5	B6	C
African accessions																
Northern Africa	38	0.413	0.256	0.681	1	4	–	–	–	5	5	13	5	5	–	–
Sudan	38	0.474	0.313	0.749	1	1	13	–	5	2	13	1	2	–	–	–
Western Africa	12	0.455	0.307	0.710	6	1	1	–	–	1	–	2	1	–	–	–
Southern Africa	9	0.434	0.325	0.621	2	–	–	6	–	–	–	1	–	–	–	–
Reference accessions																
Flexuosus	2	–	–	–	–	–	–	–	–	1	–	1	–	–	–	–
Cantalupensis	10	0.310	0.150	0.584	–	–	–	–	–	4	2	–	3	–	1	–
Inodorus	5	0.286	0.133	0.549	–	–	–	–	–	2	–	2	1	–	–	–
India	33	0.464	0.288	0.766	–	3	–	11	–	1	–	6	2	4	6	–
Myanmar	6	0.298	0.185	0.492	–	–	–	3	–	–	–	1	1	–	1	–
Vietnam	7	0.346	0.231	0.544	–	5	–	2	–	–	–	–	–	–	–	–
Conomon	4	0.164	0.156	0.179	–	4	–	–	–	–	–	–	–	–	–	–
Makuwa	5	0.211	0.147	0.320	–	5	–	–	–	–	–	–	–	–	–	–
Agrestis	5	0.379	0.267	0.571	–	2	–	1	–	–	–	1	–	–	1	–
*C. sativus* var. *hardwickii*	2	–	–	–	–	–	–	–	–	–	–	–	–	–	–	2
Total	176	0.515	0.342	0.812	10	25	14	23	5	16	20	28	15	9	9	2
Sudan-Large	17	0.235	0.066	0.524	–	–	–	–	–	2	13	1	1	–	–	–
Sudan-Small	21	0.438	0.302	0.669	1	–	14	–	5	–	–	–	1	–	–	–
India-Large	19	0.446	0.290	0.714	–	2	–	4	–	–	–	5	2	2	4	–
India-Small	14	0.432	0.238	0.764	–	–	1	7	–	1	–	1	–	2	2	–

^1^Calculated by combining RAPD and SSR data.

**Fig. 3.**
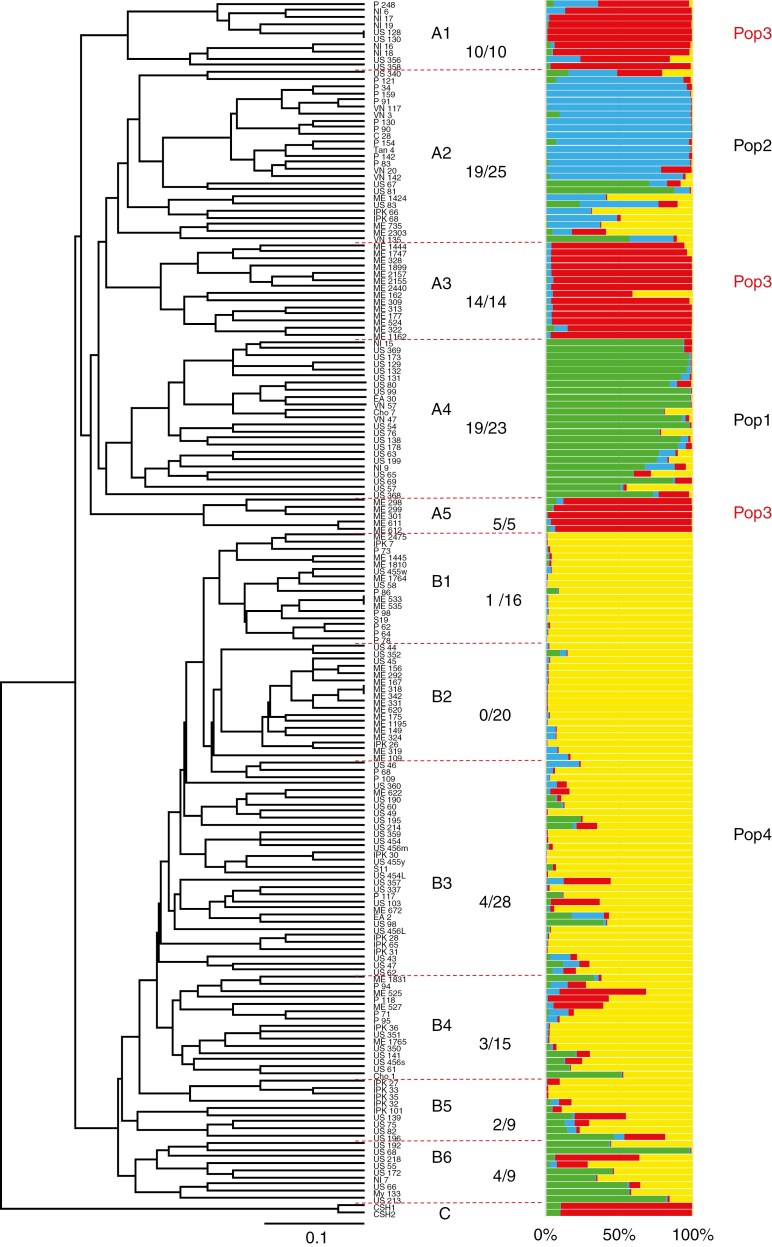
Genetic relationship between 176 accessions revealed by cluster analysis (UPGMA) based on genetic distance (left), and population structure inferred by STRUCTURE using the admixture model (right). Frequency of small seed-type in each cluster is indicated as a fraction.

The genetic relationship among 174 accessions was analysed by cluster analysis based on genetic distance (GD) calculated from the RAPD and SSR data. Melon accessions were classified into two major clusters: A and B (Fig. 3, Table 3). Cluster A included mostly small seed-type accessions (67/77) and was further divided into five subclusters. Subcluster A1 consisted of small seed-type accessions from Africa, which were frequent in Western Africa (Table 3, Supplementary Data Table S1). Subcluster A2 comprised mostly accessions from Asia, except for Myanmar, and most of them were of small seed-type. Four large seed-type accessions from Tunisia were also included. Subcluster A3 had accessions from Sudan and Western Africa, which were mostly classified as Tibish or Agrestis. Subcluster A4 consisted of accessions from Southern Africa (6/9) and South and Southeast Asia. Five Sudanese accessions with small fruits (≤60 g) formed subcluster A5.

Cluster B was further classified into six subclusters, comprising mostly large seed-type accessions (83/97) (Table 3). Accessions from Northern Africa were classified into five subclusters (B1–B5), with B3 found in all countries except for Chad and Mali (Supplementary Data Table S1). Accessions of B1 and B2 were rather frequent in Egypt (6/10), while those of B4 and B5 in Morocco and Libya (7/10), respectively. Sudanese accessions were classified into four subclusters (B1–B4), with 13 accessions in B2. Of Sudanese Flexuosus accessions, 4 were in B2, and 1 was in B3. The reference accessions of Cantalupensis and Inodorus scattered to all subclusters except B5, and Indian ssp. *melo* to all subclusters except B2. Cluster C consisted of wild cucumber accessions used as the outgroup. As indicated from these results, large seed-type accessions of Northern Africa were genetically differentiated from the small seed-type melon of Western and Southern Africa. Large and small seed-type accessions from Sudan were genetically differentiated from each other.

Through structure analysis, four major populations were hypothesized for the 174 melon accessions analysed, based on the delta *K* value (Fig. 3). All large seed-type accessions, including reference accessions of Cantalupensis, Inodorus and Flexuosus, fell into Population 4 (Pop4) or admixture with Pop4, except for four accessions from India. This result may indicate the monophyletic origin of large seed-type melons. Partial introgression from small seed-types was detected rather frequently in South Asian accessions of B6. In contrast, small seed-type accessions corresponding to cluster A were divided into three populations, indicating their polyphyletic origin. Pop1 consisted of 6 accessions from Southern Africa and 15 accessions from South and Southeast Asia (Table 4, Supplementary Data Table S1). Four large seed-type Indian accessions were also included in Pop1. Pop2 included most accessions from Vietnam, Conomon and Makuwa. Among them, Vietnamese accessions were previously classified as Conomon or Makuwa by Duong *et al.* (2021). Pop3 comprised 28 African accessions and was frequently distributed in Sudan and Western Africa. Among 22 accessions classified as admixed groups, 3 accessions from Sudan were admixture of Pop3 and Pop4 (Pop3/4), while 5 Indian and 3 Myanmar accessions were admixture of Pop1 and Pop4.

**Table 4.** Number of large and small-seed type melon accessions classified into four populations or admixture types shown in Fig. 3.

Region/group	No. of accessions	Large seed-type										Small seed-type									
		Pop 1	Pop 2	Pop 3	Pop 4	Pop 1/2	Pop 1/3	Pop 1/4	Pop 2/3	Pop 2/4	Pop 3/4	Pop 1	Pop 2	Pop 3	Pop 4	Pop 1/2	Pop 1/3	Pop 1/4	Pop 2/3	Pop 2/4	Pop 3/4
African accessions																					
Northern Africa	38	–	–	–	35	–	–	–	–	2	–	–	–	1	–	–	–	–	–	–	–
Sudan-Large	17	–	–	–	16	–	–	–	–	–	1	–	–	–	–	–	–	–	–	–	–
Sudan-Small	21	–	–	–	–	–	–	–	–	–	–	–	–	18	1	–	–	–	–	–	2
Western Africa	12	–	–	–	2	–	–	–	–	–	–	–	–	7	1	–	–	–	1	–	1
Southern Africa	9	–	–	–	1	–	–	–	–	–	–	6	–	2	–	–	–	–	–	–	–
Reference accessions																					
Flexuosus	2	–	–	–	2	–	–	–	–	–	–	–	–	–	–	–	–	–	–	–	–
Cantalupensis	10	–	–	–	9	–	–	–	–	–	1	–	–	–	–	–	–	–	–	–	–
Inodorus	5	–	–	–	4	–	–	–	–	–	1	–	–	–	–	–	–	–	–	–	–
India, large	19	4	–	–	10	–	–	5	–	–	–	–	–	–	–	–	–	–	–	–	–
India, small	14	–	–	–		–	–	–	–	–	–	9	–	–	2	1	1	–	–	–	1
Myanmar	6	–	–	–	–	–	–	–	–	–	–	3	–	–	–	–	–	3	–	–	–
Vietnam	7	–	–	–	–	–	–	–	–	–	–	2	4	–	–	1	–	–	–	–	–
Conomon	4	–	–	–	–	–	–	–	–	–	–	–	4	–	–	–	–	–	–	–	–
Makuwa	5	–	–	–	–	–	–	–	–	–	–	–	5	–	–	–	–	–	–	–	–
Agrestis	5	–	–	–	–	–	–	–	–	–	–	1	2	–	1	–	–	–	–	1	–
*C. sativus* var. *hardwickii*	2	–	–	–	–	–	–	–	–	–	–	–	–	2	–	–	–	–	–	–	–
Total	176	4	0	0	79	0	0	5	0	2	3	21	15	30	5	2	1	3	1	1	4

Classification by cluster analysis was associated well with population structure (Table 5). As for cluster A, which consists mostly of small seed-type melons, accessions from Southern Africa and South and Southeast Asia belonging to subcluster A4 fell into Pop1, while those of Conomon, Makuwa and Vietnam belonging to subcluster A2 fell into Pop2. African accessions of subcluster A1 and Sudanese accessions of subclusters A3 and A5 fell into Pop3. In contrast, accessions of cluster B fell into Pop4.

**Table 5.** Association between cluster classification, population structure, and chloroplast genome type.

Cluster	No. of accessions	No. of accessions of each population										No. of accessions of each chloroplast genome type			
		Pop1	Pop2	Pop3	Pop4	Pop 1/2	Pop 1/3	Pop 1/4	Pop 2/3	Pop 2/4	Pop 3/4	Ia	Ib	Ic	III
A1	10	–	–	10 (GA1)	–	–	–	–	–	–	–	–	–	10	–
A2	25	2	15 (GP2)	–	2	2	–	–	1	2	1	19	5	1	–
A3	14	–	–	13 (GA3)	–	–	–	–	–	–	1	1	0	13	–
A4	23	21 (GP1)	–	–	–	–	–	2	–	–	–	22	1	–	–
A5	5	–	–	5 (GA5)	–	–	–	–	–	–	–	5	–	–	–
B1	16	–	–	–	16	–	–	–	–	–	–	–	16	–	–
B2	20	–	–	–	20	–	–	–	–	–	–	–	19	1	–
B3	28	–	–	–	25	–	–	1	–	1	1	3	24	1	–
B4	15	–	–	–	12	–	–	1	–	–	2	2	11	2	–
B5	9	–	–	–	7	–	1	–	–	–	1	2	7	–	–
B6	9	2	–	–	2	–	–	4	–	–	1	7	2	–	–
C	2	–	–	2	–	–	–	–	–	–	–	–	–	–	2
Total	176	25	15	30	84	2	1	8	1	3	7	61	85	28	2

**Fig. 4.**
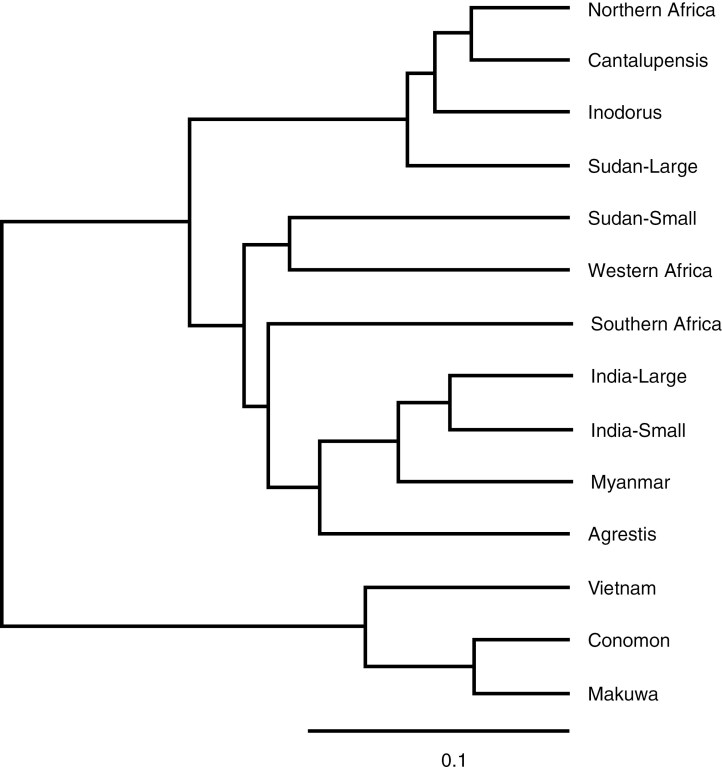
Genetic relationship among 14 geographical or horticultural groups of melon revealed by UPGMA cluster analysis based on genetic distance.

Genetic distance (GD) among 14 horticultural or geographical groups ranged from 0.073 (Conomon vs Makuwa) to 0.866 (Inodorus vs Makuwa), and from 0.123 (Northern Africa vs large seed-type from Sudan) to 0.478 (large seed-type vs small seed-type from Sudan) among 5 groups of African melon (Supplementary Data Table S5). Fourteen groups were separated into three clusters. Small seed-type accessions from Sudan were clustered together with Western Africa, where 10 of 12 accessions were small seed-types (Fig. 4). These two groups were also close to Southern Africa, Agrestis, Myanmar and India. In contrast, large seed-type accessions from Sudan clustered together with Northern Africa, where 37 of the 38 accessions were large seed-type, as well as with Cantalupensis and Inodorus. Accessions from Vietnam and of Conomon and Makuwa were distantly related to other groups. The genetic relationship among geographical or horticultural groups mentioned above was also reproduced by principal coordinate analysis (Supplementary Data Fig. S3).

### Analysis of chloroplast genome markers

Cytoplasm type, which differed among maternal lineages, was determined by the analysis of three diagnostic markers of the chloroplast genome, and 61, 85 and 28 accessions were assigned as Ia, Ib and Ic types, respectively (Table 6, Supplementary Data Table S4). Most of the large seed-type accessions, including Cantalupensis, Inodorus and Flexuosus, were Ib type. In contrast, small seed-type accessions consisted mostly of two maternal lineages, with 51 and 26 out of 81 accessions being Ia and Ic types, respectively. Ic type was found only in Africa, and 92.9 % of Ic type accessions were small seed-type. Ia type was frequently found in Sudan and Southern Africa as well as South, Southeast and East Asia. Small seed-type accessions from Sudan and Southern Africa proved to be a mixture of Ia and Ic lineages.

## DISCUSSION

Although Africa has long been considered the centre of melon domestication (Robinson and Decker-Walters, 1997), our knowledge about the diversity of African melon is still very limited and fragmented (Mliki *et al.*, 2001; El-Tahir and Yousif, 2004; Tanaka *et al.*, 2013, 2024; Trimech *et al.*, 2013, 2015; Koffi *et al.*, 2014; Imen *et al.*, 2015; Wang *et al.*, 2021). To understand the genetic structure of African melon, in this study genetic diversity was analysed for 97 accessions collected from 16 countries in Africa (Supplementary Data Table S1). Abundant genetic diversity equivalent to that of the Indian melon analysed in this study was confirmed in four geographical groups of Africa: Northern, Western and Southern Africa and Sudan (Table 3). Gene diversity in all African accessions was high (*D* = 0.508; calculated from RAPD and SSR data). Shigita *et al.* (2023) analysed the genetic diversity of 755 melon accessions with diverse geographical origins by genotyping-by-sequencing and established a world core collection of 100 accessions. Of these, 21 and 23 were of African and Indian origin, respectively, indicating abundant genetic diversity in these areas. Considering the presence of primitive (or wild) types of melon in India (Dhillon *et al.*, 2007; John *et al.*, 2013) and the very limited exploration of African germplasms, genetic diversity in these areas is considered underestimated.

**Fig. 5.**
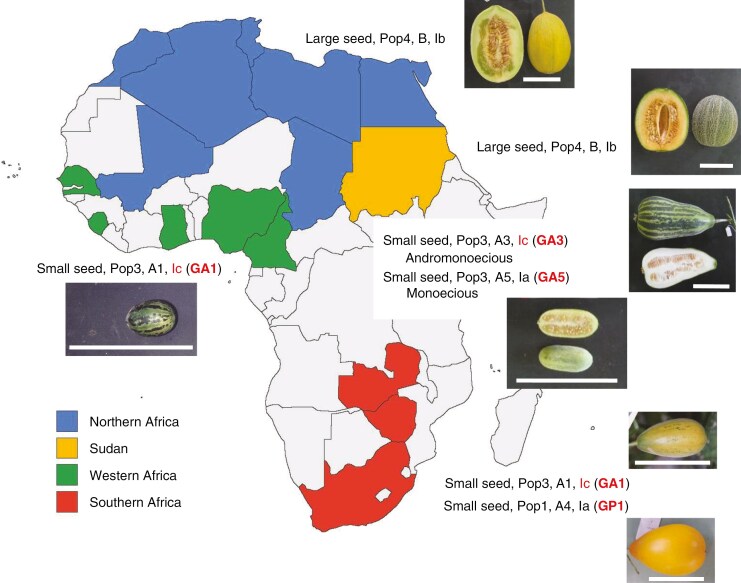
Allopatric distribution of three lineages of melon in Africa. The type of melon predominant in each area is indicated in the following order: seed size, population (structure analysis), cluster (UPGMA cluster analysis), and chloroplast genome type. Fruit photograph of a representative accession is also shown. Scale bars are 10 cm.

Both large and small seed-type accessions are commonly found in the African continent, but their frequency showed distinct geographical differences (Table 1, *χ*^2^ = 43.67, d.f. = 3, *P* < 0.01). In good accordance with Wang *et al.* (2021) and Tanaka *et al.* (2024), accessions from Northern Africa were mostly large seed-types, while small seed-types were frequently found in Western and Southern Africa (Fig. 5). Hence, Northern Africa is rich in genetic diversity of large seed-type melons, while Western and Southern Africa are rich in genetic diversity of small seed-type melons. In Sudan, both large and small seed-types are commonly cultivated (Table 1).

Tanaka *et al.* (2013, 2024) revealed the presence of three maternal lineages (Ia, Ib and Ic) in melon. Lineage Ia contained small seed-type melons from Southern Africa and South and East Asia, and lineage Ib consisted of large seed-type melons from Northern Africa, Europe and the USA. In contrast, lineage Ic is limited to Western and Southern Africa. This result was confirmed by resequencing of the chloroplast genome of 977 melon accessions (Zhao *et al.*, 2019), and a similar result was presented by the analysis of partial sequences of the nuclear ribosomal ITS and chloroplast genome (Endl *et al.*, 2018). In this study, large seed-type accessions from Northern Africa and Sudan belonged to lineage Ib (Table 6). Small seed-type accessions of lineage Ia were common in Sudan and Southern Africa, and those of Ic were common in Sudan and Western and Southern Africa. These results clearly showed the allopatric distribution of the three lineages of melon in Africa (Fig. 5), closely related to seed size type (Table 6) and the population structure of the nuclear genome (Table 4).

Small seed-type accessions were separated into the following five groups based on the polymorphisms in the nuclear and chloroplast genomes (Table 5, Fig. 5): (1) (GP1) Pop1, A4 and Ia (South and Southeast Asia and Southern Africa); (2) (GP2) Pop2, A2 and Ia (Conomon, Makuwa); (3) (GA1) Pop3, A1 and Ic (Africa, frequent in Western Africa); (4) (GA3) Pop3, A3 and Ic (mostly Sudan); and (5) (GA5) Pop3, A5 and Ia (Sudan). In all five groups, accessions of Agrestis with small fruit (≤60 g), which were considered non-cultivated feral or wild melon (Pitrat, 2016), were detected. Among 26 small-fruited accessions including 5 reference accessions of Agrestis, 4 and 2 from Asia belonged to groups GP1 and GP2, respectively, while 5, 4 and 5 from Africa belonged to groups GA1, GA3 and GA5, respectively. Therefore, in four groups except for GA5, small-fruited Agrestis accessions were included in the same genetic group with cultivated melon in each area, although further studies are required to conclude which is feral or wild. Except for group GA5, small seed-type melons could be roughly divided into two geographical groups: Asian with Ia cytoplasm (groups GP1 and GP2) and African with Ic cytoplasm (groups GA1 and GA3), supporting the polyphyletic origin of cultivated melon (Esteras *et al.*, 2013; Pitrat, 2013; Tanaka *et al.*, 2013, 2024; Endl *et al.*, 2018). The introduction of melon from Asia to Southern Africa (Nakata *et al.*, 2005; Zhao *et al.*, 2019; Wang *et al.*, 2021) was also confirmed by the presence of group GP1 accessions in Southern Africa. The two genetic groups of melon in Southern Africa reported by Wang *et al.* (2021) correspond to group GP1 (Ia lineage) introduced from South Asia and group GA1 (Ic lineage) introduced from the north (Tables 4 and 6).

In Sudan, genetic diversity was clearly different among large and small seed-types, being high in small seed-types and low in large seed-types (Table 3). Large seed-type accessions of Sudan belonged to cluster B (Table 3), Pop4 (Table 4) and Ib maternal lineage (Table 6), along with other accessions of large seed-type melons, and contrastingly different from Sudanese small seed-type accessions (A3 or A5, Pop3 and Ia or Ic). Genetic differences among large and small seed-types in Sudan were also shown by large GD (0.478) between the two populations (Supplementary Data Table S5). These results clearly demonstrate the coexistence of melon populations with different histories in Sudan, supporting the statement of El-Tahir and Yousif (2004) that traditional cultivars of both sweet and snake melon are old introductions from other regions, while Tibish and wild Agrestis are truly indigenous to this region. The introduction of large seed-type melons from outside Sudan can be confirmed by genetic similarity with large seed-type accessions from other areas, including India. From another standpoint, recombinants such as the large seed-type of cluster A or Ia cytoplasm type were very common in India, while they were rarely found in Sudan (Table 3). Since melon is an allogamous plant species, hybrids between two types should often be produced in the field, as also suggested by Tanaka *et al.* (2024). Therefore, it was suggested that small seed-type melons were originally grown and utilized as a vegetable crop in Sudan and large seed-type melons were introduced later as a kind of different ‘crop’, and that local people consciously selected to retain the traits of each ‘crop’. In this context, Sudanese melon accessions were clearly classified into four groups based on their horticultural traits (Fig. 2, Table 2), and this classification was closely associated with genetic classification (Fig. 2, Supplementary Data Table S6), suggesting that spontaneous hybrids between both ‘crops’ were removed by conscious selection.

Small seed-type Sudanese accessions proved to be a mixture of lineages Ia (group GA5) and Ic (mostly group GA3), as mentioned above (Table 5). Accessions of vegetable melon Tibish, unique to Sudan, belonged to group GA3, together with small-fruited Agrestis accessions (feral or wild). In contrast, all five accessions of group GA5 were feral or wild with small fruits, and cultivated melon was not included in this group. Two accessions (CUM 287 and PI 185111) used in this study were classified as *C. melo* ssp. *meloides* based on sequence polymorphism at seven chloroplast regions and one nuclear region by Endl *et al.* (2018). Of these, CUM 287 belonged to group GA3 together with Tibish accessions. When groups or taxa are defined by some molecular markers it is very difficult to assign an accession to such a group or taxon when other markers or traits are used. Nevertheless we can reasonably think that group GA3, not GA5, corresponds to spp. *meloides*; further studies including morphological characterization are awaited. The flower type of GA5 accessions was monoecious, while that of GA3 accessions, including Tibish, was andromonoecious. Therefore, the possible candidate for the ancestor of Tibish was not the feral or wild type of group GA5 but that of group GA3. Among the 13 accessions of group GA3, four were small-fruited and showed variation in flower type, being andromonoecious (ME 2440), monoecious (ME 1444, ME 1747) and segregating (ME 328). The andromonoecious type of feral or wild melon is not common in general and might be derived from hybridization between Tibish and the monoecious type of feral or wild melon. Alternatively, andromonoecy might have arisen by mutation in the feral or wild type of group GA3, and thereafter Tibish might have been established through the selection of larger and non-bitter fruits. In this connection, Tibish was reported to have a unique genetic mechanism for andromonoecy (Abdelmohsin *et al.*, 2012), which is different from that regulated by the *CmACS-7* gene (Boualem *et al.*, 2008). Therefore, the genetic mechanism for andromonoecy should be clarified in Tibish.

Another candidate for the ancestor of Tibish might be the feral or wild type of group GA1, which is distributed widely in Africa (Fig. 5). Six of the ten accessions of group GA1 were selected for the core collection by Shigita *et al.* (2023). According to Wang *et al.* (2021), nine accessions from Africa were positioned close to the outgroup. Of these nine accessions, five accessions were commonly used in this study, and three fell into GA1. These results indicate abundant and unique genetic diversity in GA1. Chevalier (1901) conducted a field trip in the ‘western Sudan region’, currently the southwestern part of Mali, and stated, ‘In western Sudan, and especially in the Middle Niger Valley, the *Cucumis melo* abounds during winter from July to November, in the savannas, on the grassy terraces of the rocks, and in the turfed sands near the waters. He would be in the middle of the bush in such great abundance and in such conditions that it is impossible to doubt his spontaneity’. Taking these findings together, it could be reasonably hypothesized that the melon of Ic lineage, including Tibish, originated from wild melon in the ‘western Sudan region’, independently of melon of Ia or Ib lineages originating in Asia. The geographically limited distribution of Ic type melon may suggest its recent origin compared with Ia and Ib type melons. *Cucumis melo* var. *cossonianus* (Chevalier, 1901) or wild type of ssp. *meloides* (El-Tahir and Pitrat, 1999; Endl *et al.*, 2018) can be a possible wild ancestor of Ic type melon. To uncover the origin and domestication of Ic type melon, intensive field studies should be conducted in the ‘western Sudan region’, and molecular genetic studies should be carried out for the genetic resources collected, including Humaid, known as wild Agrestis (El-Tahir and Yousif, 2004).

## SUPPLEMENTARY DATA

Supplementary data are available at *Annals of Botany* online and consist of the following. Table S1: melon accessions analysed in this study. Table S2: primer sequences of 12 RAPD and 7 SSR markers used in this study and the size of polymorphic fragments. Table S3: three markers used for the analyses of chloroplast genome type. Table S4: sequence differences among three chloroplast genome types. Table S5: genetic distance among five groups of African melon and nine reference groups. Table S6: association between morphological classification and molecular classifications. Figure S2: similarity and dissimilarity in morphological traits among 46 accessions of melon revealed by PCA. Figure S1: gel profile of African melon landraces. Figure S3: genetic relationship among 14 geographical or horticultural groups of melon shown on the first two principal coordinates.

mcaf028_suppl_Supplementary_Tables_S1

mcaf028_suppl_Supplementary_Tables_S2

mcaf028_suppl_Supplementary_Tables_S3

mcaf028_suppl_Supplementary_Figures_S1

mcaf028_suppl_Supplementary_Tables_S4

mcaf028_suppl_Supplementary_Figures_S2

mcaf028_suppl_Supplementary_Tables_S5

mcaf028_suppl_Supplementary_Figures_S3

mcaf028_suppl_Supplementary_Tables_S6
