# Supplementary material for: Molecular polymorphisms of the nuclear and chloroplast genomes among African melon germplasms reveal abundant and unique genetic diversity, especially in Sudan
Source: Ann Bot. 2025 Apr 17;135(7):1329–44. doi: 10.1093/aob/mcaf028 (PMC12358025; doi:10.1093/aob/mcaf028)
Supplement: mcaf028_suppl_Supplementary_Figures_S1 [file mcaf028_suppl_supplementary_figures_s1.pptx]

## Slide 1
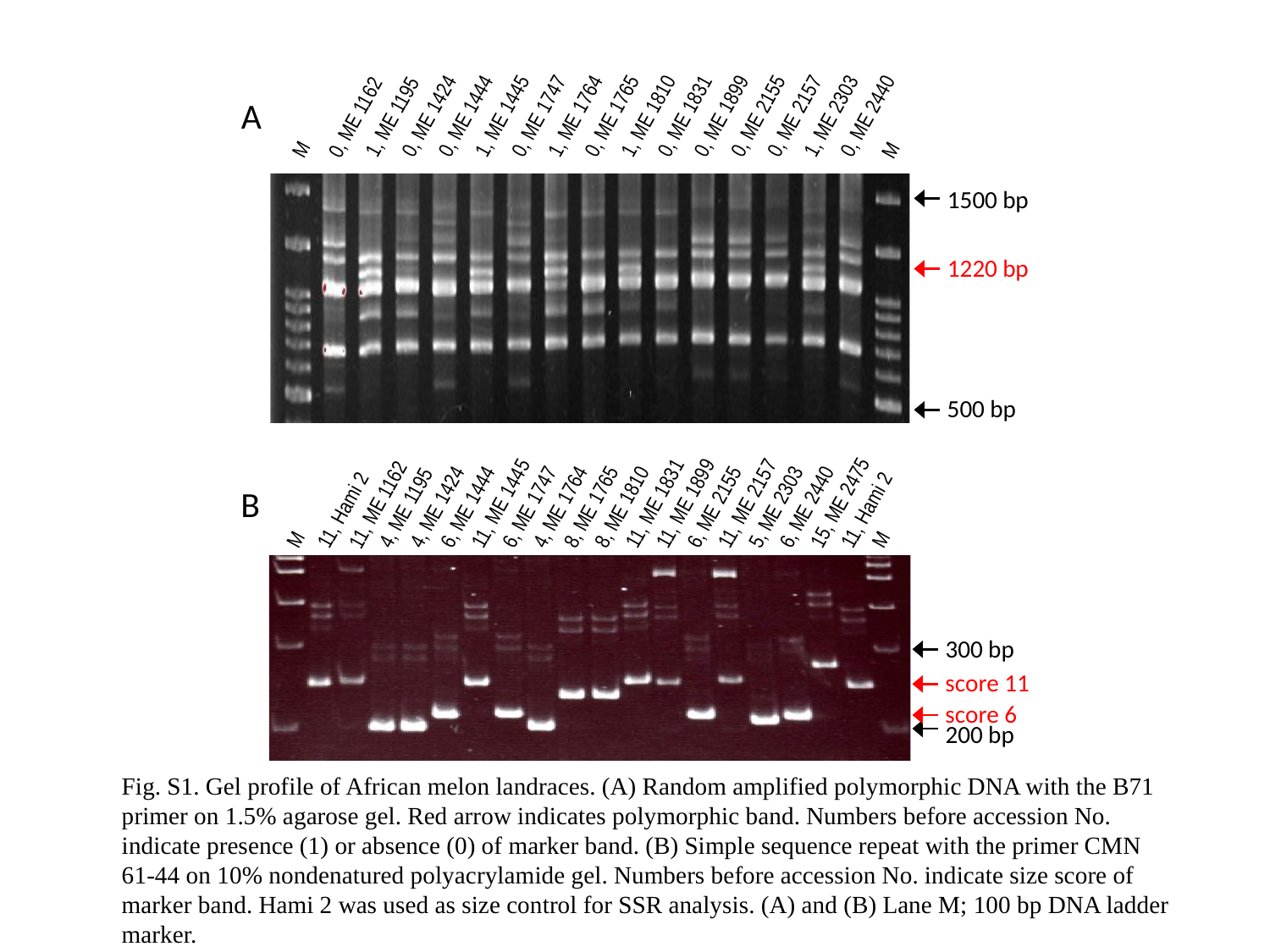

A
1, ME 1195
0, ME 1424
0, ME 1444
1, ME 1445
0, ME 1747
1, ME 1764
0, ME 1765
1, ME 1810
0, ME 1831
0, ME 1899
0, ME 2155
0, ME 2157
1, ME 2303
0, ME 2440
0, ME 1162
M
M
1500 bp
1220 bp
500 bp
B
11, ME 1445
15, ME 2475
11, ME 2157
11, ME 1899
11, ME 1831
M
11, Hami 2
4, ME 1195
4, ME 1424
6, ME 1444
6, ME 1747
4, ME 1764
8, ME 1765
8, ME 1810
6, ME 2155
5, ME 2303
6, ME 2440
11, Hami 2
M
11, ME 1162
300 bp
score 11
score 6
200 bp
Fig. S1. Gel profile of African melon landraces. (A) Random amplified polymorphic DNA with the B71 primer on 1.5% agarose gel. Red arrow indicates polymorphic band. Numbers before accession No. indicate presence (1) or absence (0) of marker band. (B) Simple sequence repeat with the primer CMN 61-44 on 10% nondenatured polyacrylamide gel. Numbers before accession No. indicate size score of marker band. Hami 2 was used as size control for SSR analysis. (A) and (B) Lane M; 100 bp DNA ladder marker.
