# Supplementary material for: Molecular polymorphisms of the nuclear and chloroplast genomes among African melon germplasms reveal abundant and unique genetic diversity, especially in Sudan
Source: Ann Bot. 2025 Apr 17;135(7):1329–44. doi: 10.1093/aob/mcaf028 (PMC12358025; doi:10.1093/aob/mcaf028)
Supplement: mcaf028_suppl_Supplementary_Figures_S2 [file mcaf028_suppl_supplementary_figures_s2.pptx]

## Slide 1
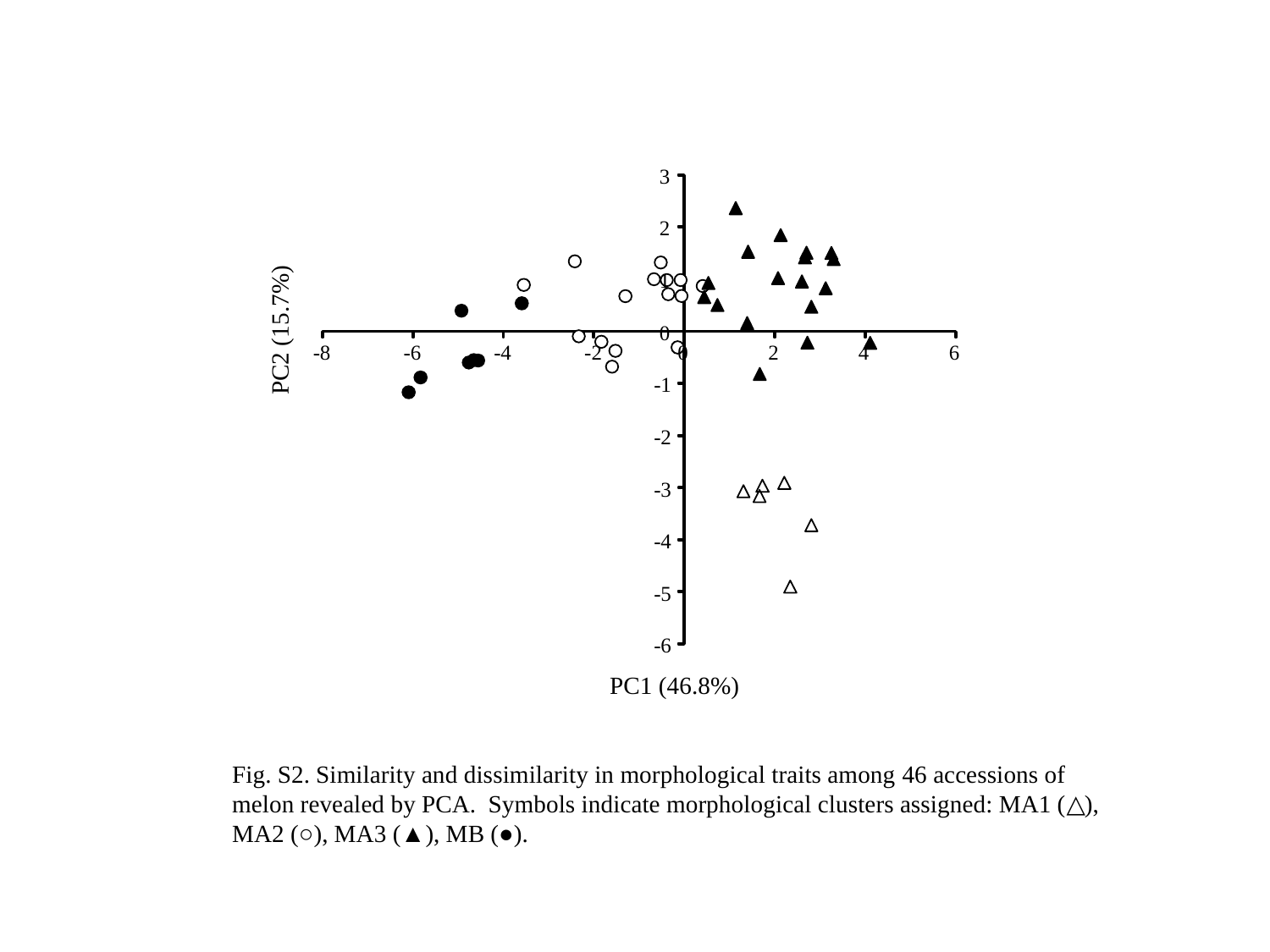

3
2
1
0
-1
-2
-3
-4
-5
-6
-8
-6
-4
-2
0
2
4
6
PC2 (15.7%)
PC1 (46.8%)
Fig. S2. Similarity and dissimilarity in morphological traits among 46 accessions of melon revealed by PCA. Symbols indicate morphological clusters assigned: MA1 (△), MA2 (○), MA3 (▲), MB (●).
