# Supplementary material for: Molecular polymorphisms of the nuclear and chloroplast genomes among African melon germplasms reveal abundant and unique genetic diversity, especially in Sudan
Source: Ann Bot. 2025 Apr 17;135(7):1329–44. doi: 10.1093/aob/mcaf028 (PMC12358025; doi:10.1093/aob/mcaf028)
Supplement: mcaf028_suppl_Supplementary_Figures_S3 [file mcaf028_suppl_supplementary_figures_s3.pptx]

## Slide 1
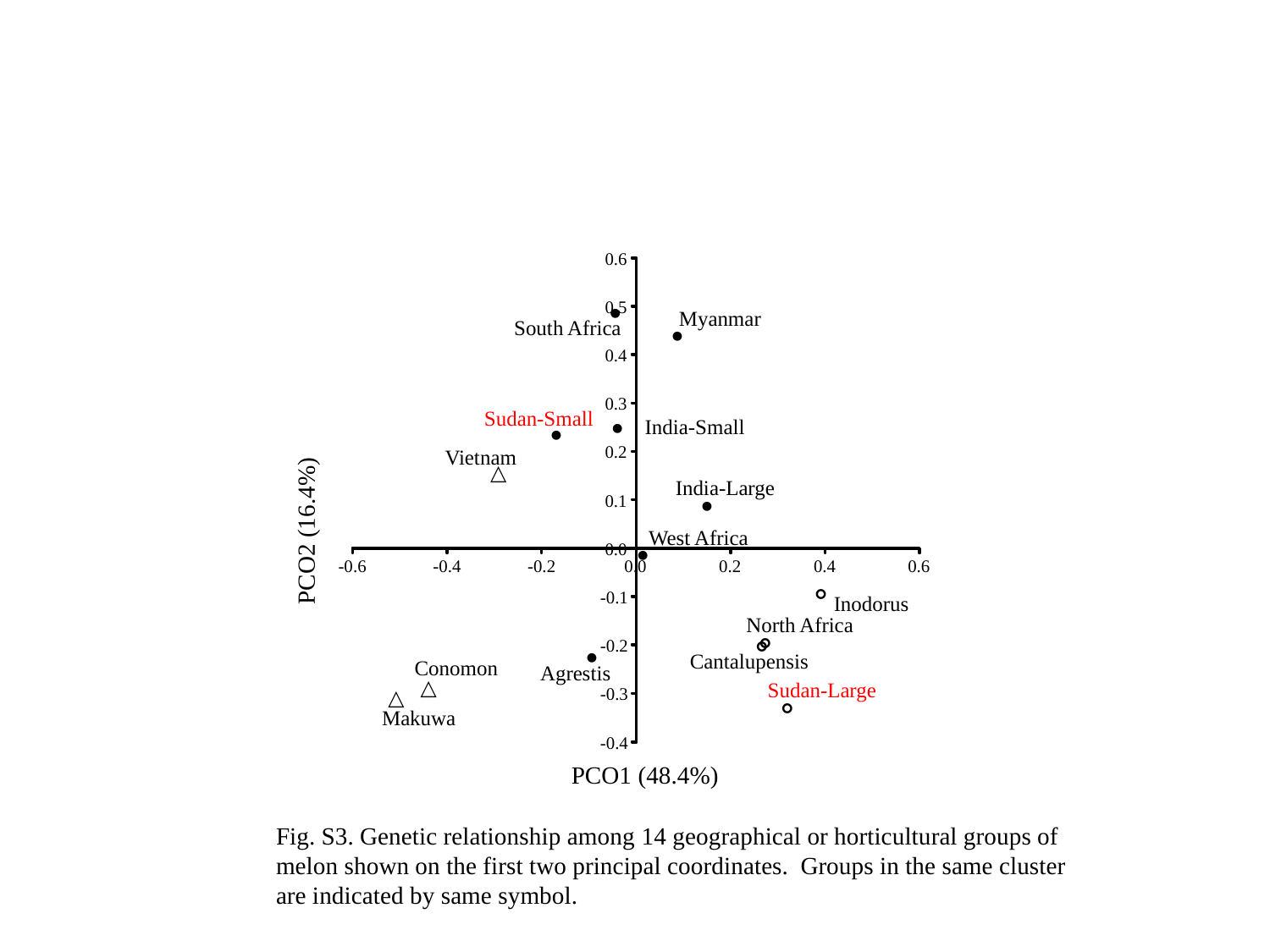

0.6
0.5
0.4
0.3
0.2
0.1
0.0
-0.1
-0.2
-0.3
-0.4
●
Myanmar
South Africa
●
Sudan-Small
●
India-Small
●
Vietnam
△
India-Large
●
West Africa
●
-0.6
-0.4
-0.2
0.0
0.2
0.4
0.6
○
Inodorus
North Africa
○
○
●
Cantalupensis
Conomon
Agrestis
△
Sudan-Large
△
○
Makuwa
PCO2 (16.4%)
PCO1 (48.4%)
Fig. S3. Genetic relationship among 14 geographical or horticultural groups of melon shown on the first two principal coordinates. Groups in the same cluster are indicated by same symbol.
